# 10.P. Round table: Public health of the future: innovations in surveillance, communication and knowledge translation

**DOI:** 10.1093/eurpub/ckac129.680

**Published:** 2022-10-25

**Authors:** Organised by: SESPAS, EUPHA-PHPP, ASPHER, Chair persons: Enrique Bernal-Delgado (Spain), Gabriela Barbaglia (Spain)

## Abstract

The social, ecological, economic and health crises exacerbated by COVID-19 pandemic are challenges of extraordinary magnitude and complexity for global public health. Moreover, the context in which the pandemic emerged was characterized by underinvestment in public health and growing distrust in institutions. Public health responses were often fragmented and failed to make use of existing resources and expertise. Nearly 3 years after the start of the COVID-19 pandemic much has been learned and much is still to be learned. Accordingly, European national public health agencies have been pushed to their limits and currently face an urgent need to be renovated incorporating innovations in surveillance, communication and knowledge translation. National agencies should network and collaborate at the EU level. EUPHA may play an important role in this effort.

On the one hand, there is the need of improving surveillance of harmful effects of the pandemic, specifically the health inequalities aggravated at local, national and global levels; and, on the other, to improve the availability of this knowledge to policymakers. Public Health communication needs to be further developed as it has been a crucial piece of national and international efforts to protect and promote health in the pandemic and so will be in the future. With this workshop proposal, we would like to bring up for discussion how could we further improve surveillance, communication and knowledge translation to policy makers and citizens in our European national public health agencies. Innovative and updated public health agencies will help regaining trust and strengthening public health institutions. National and European Public Health further development is essential and should be strengthened to protect and promote European populatiońs health.

The objectives of the workshop are:

– To discuss key innovations to implement in national public health agencies to improve surveillance, communication and knowledge transfer to policy makers and citizens.

– To reflect on supranational European coordination mechanisms that would allow for efficient surveillance and a rapid and adequate response to different public health challenges, including social inequalities in health.

– To manage public health intelligence in the European Health Data Space and the role of public health in this data lake design.

**Key messages:**

• COVID19 pandemic has revealed the challenges of creating strong trustworthy national public health institutions to ensure the integrity of public health science and information dissemination.

• Structures to facilitate timely and efficient monitoring requires national and supranational coordination mechanisms, including data and experience sharing.

**Speakers/Panellists:**

**Robert Otok**

ASPHER, Brussels, Belgium

**Sofia Ribeiro**

EUPHA-PHPP

**Manuel Franco**

University of Alcalá, Madrid, Spain

